# Effect of Biobased and Mineral Additives on the Properties of Recycled Polypropylene Packaging Materials

**DOI:** 10.3390/polym17172368

**Published:** 2025-08-30

**Authors:** Wiktor Wyderkiewicz, Robert Gogolewski, Justyna Miedzianowska-Masłowska, Konrad Szustakiewicz, Marcin Masłowski

**Affiliations:** 1 Institute of Polymer and Dye Technology, Lodz University of Technology, Stefanowskiego 16, 90-537 Lodz, Poland; 254034@edu.p.lodz.pl (W.W.); justyna.miedzianowska@p.lodz.pl (J.M.-M.); 2 CDM Packaging, Cegielniana 7, 95-054 Ksawerów, Poland; robert.gogolewski@cdm.pl; 3 Department of Polymer Engineering and Technology, Faculty of Chemistry, Wroclaw University of Science and Technology, Wybrzeże Wyspiańskiego 27, 50-370 Wrocław, Poland; konrad.szustakiewicz@pwr.edu.pl

**Keywords:** recycling, packaging materials, polypropylene foils, biochar, diatomaceous earth, composites, functional properties

## Abstract

The recycling of polypropylene (PP) packaging films modified with biobased additives: biochar derived from the pyrolysis of natural fibers and diatomaceous earth was investigated. The aim was to assess the impact of these modifiers on the processing, rheological, mechanical, and thermal properties of the recycled material. The processing behavior was evaluated through extrusion with granulation to determine industrial applicability. Rheological properties, including viscosity and melt flow index (MFI), were measured to characterize flow behavior. Mechanical performance was assessed through tensile strength, hardness, three-point bending, and impact resistance tests. Thermal properties were analyzed using thermogravimetric analysis (TGA), Vicat softening temperature (VST), and differential scanning calorimetry (DSC). The results demonstrate that incorporating biochar and diatomaceous earth can modify and, in selected cases, enhance the processing and performance characteristics of recycled PP films, though their impact on thermal behavior is parameter-specific. While diatomaceous earth slightly increased the onset of thermal degradation (T_5_), both fillers caused a slight decrease in the VST, indicating reduced heat resistance under load. Diatomaceous earth was found to effectively improve stiffness and impact strength, while biochar reduced viscosity and promoted finer crystalline structures. Both additives acted as nucleating agents, increasing crystallization temperatures, with diatomaceous earth additionally delaying thermal degradation onset. These findings highlight the potential of using sustainable, waste-derived additives in polymer recycling, supporting the development of environmentally responsible materials within circular economy frameworks.

## 1. Introduction

The escalating production and consumption of plastics worldwide have led to an alarming increase in plastic waste, posing severe environmental challenges [1]. Among plastics, polypropylene (PP) is one of the most widely used polymers, especially in packaging applications, due to its favorable mechanical properties, chemical resistance, low cost, and ease of processing [2]. However, polypropylene films, often utilized in flexible packaging, are typically discarded after a single use, contributing significantly to environmental pollution. Addressing this issue requires effective recycling strategies combined with innovative approaches to enhance the performance of recycled materials [3]. Mechanical recycling of polypropylene films converts post-consumer or post-industrial waste into secondary raw materials, reducing landfill disposal and resource depletion [2,4]. However, recycled polypropylene frequently suffers from deterioration in mechanical and thermal properties caused by polymer chain degradation, contamination, and poor filler dispersion [5]. Therefore, improving the performance of recycled polypropylene composites is a critical research focus [6]. One promising approach to upgrading recycled polypropylene involves the incorporation of biobased fillers, which not only enhance material properties but also contribute to sustainability by valorizing biomass waste [5]. Biochar and diatomaceous earth (DE) are two distinct biofillers garnering attention due to their availability, environmental friendliness, and ability to improve polymer composite performance [7].

Recycled polypropylene films were utilized as matrices for biocomposites reinforced with biochar or diatomaceous earth, applied separately in different formulations. It discusses the recycling process of polypropylene films, including processing and preparation techniques, the fundamental characteristics and roles of biochar and diatomaceous earth as biofillers, and their impacts on the properties of the resulting composites. This study aimed to highlight how these biofillers can contribute to the development of sustainable, high-performance composite materials.

Biofillers derived from renewable biomass sources are particularly attractive because they enhance sustainability and contribute to circular economy models by converting agricultural or industrial by-products into value-added materials [5]. In this context, biochar and diatomaceous earth have been identified as effective fillers, providing distinct advantages based on their composition and morphology [8,9].

Biochar is a carbon-rich solid produced through the pyrolysis of biomass under limited oxygen conditions. This thermochemical conversion preserves much of the carbon content while generating a porous, high-surface-area material rich in aromatic carbon structures and functional groups such as hydroxyl, carboxyl, and carbonyl [10,11]. These characteristics make biochar an excellent biofiller for polymer composites, including recycled polypropylene. The porous nature of biochar facilitates mechanical interlocking and improved adhesion between the filler and polymer matrix, enhancing stress transfer and overall mechanical strength [5,12]. Studies have shown that biochar incorporation increases the tensile strength, Young’s modulus, and impact resistance of polypropylene composites, mitigating the loss of properties typically associated with recycled polymers [10]. Thermal stability is also significantly improved by biochar addition. Due to its inherent high thermal resistance and ability to act as a heat sink, biochar-filled composites display higher degradation onset temperatures and enhanced flame retardancy [11]. Furthermore, biochar particles can serve as nucleating agents, promoting faster and more uniform crystallization of polypropylene, which results in improved dimensional stability and mechanical properties [5,13]. The environmental benefits of using biochar are notable. It contributes to carbon sequestration by locking carbon in a stable form, reducing greenhouse gas emissions when used in polymer composites [11]. Additionally, biochar is produced from various biomass wastes, including agricultural residues, forestry by-products, and food industry wastes, thus supporting waste valorization and resource efficiency [14].

Diatomaceous earth (DE) is a naturally occurring sedimentary mineral composed primarily of fossilized diatom frustules, which are microscopic algae shells made of amorphous silica. DE is characterized by its highly porous, fine particulate morphology and a large specific surface area [15]. These properties make DE an attractive biofiller for reinforcing recycled composites. When incorporated into matrices, DE acts as a rigid filler that restricts polymer chain mobility, thereby increasing stiffness and hardness [5,16]. This effect translates into improved mechanical properties, including an enhanced modulus and surface durability. The thermal stability of polypropylene composites is also enhanced by DE due to its high thermal resistance and ability to dissipate heat efficiently [15,17]. Beyond mechanical and thermal improvements, DE contributes to better barrier properties in packaging materials by reducing permeability to gases and moisture. The porous structure and tortuous path created by DE particles impede the diffusion of oxygen and water vapor, which is critical for food packaging applications aimed at extending product shelf life [15,18]. Additionally, DE is abundant, cost-effective, and non-toxic, presenting a sustainable alternative to conventional mineral fillers such as talc or calcium carbonate [19,20]. While DE incorporation yields many benefits, potential drawbacks include increased brittleness at higher loadings and processing challenges due to increased composite viscosity [21]. Careful optimization of filler content and processing conditions is necessary to balance these effects.

The integration of recycled polypropylene packaging films with biofillers such as biochar and diatomaceous earth presents a promising avenue for developing sustainable, high-performance biocomposites tailored for various applications [22]. Recycling polypropylene reduces plastic waste and mitigates environmental impacts, while the addition of biobased fillers compensates for the mechanical and thermal property losses typically associated with recycled materials [23,24]. The synergistic combination of polypropylene film recycling and biofiller reinforcement aligns with global sustainability goals by promoting circular economy principles, reducing reliance on fossil resources, and enabling the development of environmentally responsible composite solutions [25,26].

## 2. Materials and Methods

### 2.1. Polypropylene Matrix

Post-industrial cutting waste of biaxially oriented polypropylene (BOPP) film was supplied by CDM Packaging (Poland). We differentiated two waste streams: a transparent, unprinted film (PP-CL) and the identical film printed with flexographic ink for apple-packaging applications (PP-PR).

### 2.2. Biofillers

Two natural fillers were employed in this experimental work. Biochar (BC) was produced in three successive steps. First, dried cereal straw was coarsely ground in a laboratory ball mill (FRITSCH PULVERISETTE 5, Idar-Oberstein, Germany) to obtain a homogeneous, coarse powder. The material was then comminuted further in a planetary mill (SPEX SamplePrep 8000D Mixer/Mill, Metuchen, NJ, USA), which markedly reduced the particle size and yielded a fine fraction. The biochar particles exhibited a fibrous–porous structure with heterogeneous dimensions, ranging from a few micrometers up to several hundred micrometers.

In the final step, the pulverized straw underwent pyrolysis under an inert atmosphere. The material was placed in a LIFT 3.0 BT furnace (NEOTERM Electromechanical Plant, Wroclaw, Poland) and heated to 600 °C for 2 h, followed by cooling in a continuous nitrogen stream to prevent oxidation of the freshly formed biochar.

The mineral biofiller used was a ready-to-use, non-calcined diatomite marketed under the NANGA brand (1 kg; food-grade quality). The material was supplied as a fine, homogeneous powder free of visible agglomerates and, therefore, required no additional pre-treatment. After a brief visual inspection, the powder was weighed to the prescribed mass, added directly to the polypropylene matrix, and mechanically premixed prior to the subsequent extrusion step.

### 2.3. Composition

The formulations of the polypropylene (PP) composites are summarized in Table 1. All recipes are expressed per 100 parts by weight (pbw) of PP, which serves as the reference basis (PP = 100 pbw). Where applicable, 5 pbw of a single biofiller—either biochar (BC) or diatomaceous earth (DE)—was incorporated. This loading corresponded to 4.8 wt % of filler in the finished composite. Two matrix variants were examined, neat PP and printed PP, giving six materials in total.

### 2.4. Preparation of Polypropylene Pre-Compounds with Biofillers

The fabrication of the composites began with mechanical size reduction of post-consumer polypropylene (PP) film. Sheets of unprinted and flexographically printed film were manually cut with scissors into small fragments to facilitate feeding into the extrusion hopper.

The shredded material was processed in a laboratory ZAMAK Mercator single-screw extruder (Skawina, Poland) equipped with three heating zones set to 180 °C, 190 °C, and 200 °C, respectively. The first zone plasticized the feed, the second promoted melt homogenization and devolatilization, and the third stabilized the melt pressure before the strand was discharged. The hot extrudate was conveyed along a cooled belt fitted with a stabilizing roller and subsequently pelletized in a Brabender strand granulator (Duisburg, Germany) to obtain a uniform re-granulate.

### 2.5. Injection Molding of Polypropylene Composites with Biofillers

Mechanical test specimens were produced by injection molding with a Battenfeld PLUS 350 machine (Kottingbrunn, Austria). Processing began with the reference granulates (neat PP and printed PP), after which the composite masterbatches containing 4.8 wt % biochar or diatomaceous earth were molded in the same order. Each formulation was charged to the hopper separately to eliminate cross-contamination, and the machine barrel was purged between runs.

The press was operated in manual mode using a five-step cycle: the mold was first closed, the plastifying unit was retracted to admit material and then advanced, followed by polymer injection with a brief holding pressure stage, and finally mold opening and ejection of the finished part. All materials were processed with a two-zone temperature profile—210 °C in the barrel and 220 °C at the nozzle—which corresponds to the recommended processing window for polypropylene. Freshly molded dumbbell and bar specimens thus obtained were subsequently conditioned for mechanical testing.

### 2.6. Hardness

Shore D hardness was determined in accordance with PN-EN ISO 868 using a Zwick 3105 durometer (Ulm, Germany) fitted with a 30° conical indenter (tip radius: 0.1 mm) designed for rigid thermoplastics and resins. Standard injection-molded bar specimens from each formulation were conditioned and tested under ambient laboratory conditions. For every bar, ten determinations were made—five on each face—while respecting the 5 s dwell time specified in the standard. The hardness reported for a given material represents the arithmetic mean of the ten individual readings.

### 2.7. Impact Strength

The resistance of the PP composites to dynamic fracture was assessed by the Charpy method in accordance with PN-EN ISO 179-1 [27]. Tests were conducted with a Cometech QC-639P (New Taipei City, Taiwan) pendulum impact tester. Before each test, the thickness of the bar was measured with a digital caliper (resolution: 0.01 mm) at several points, and the minimum value (*h*_min_) was adopted for the calculations, as recommended by the standard to avoid overestimating the impact strength.

Specimens were positioned on the supports of the pendulum rig and struck by the Charpy hammer. For every formulation, four impacts were performed, and the absorbed energy *E*_s_ [J] displayed by the instrument was recorded. The specific absorbed energy *E*_sa_ [kJ/m^2^] was calculated with

(1) $$E_{sa}=\frac{E_{s}}{b\times h_{min}}\times 10^{3}$$

where

$E_{sa}$—the specific absorbed energy [kJ m^−2^];

$E_{s}$—the energy absorbed during impact [J];

*b*—the specimen width at the impact plane [mm];

$h_{min}$—the minimum measured specimen thickness [mm];

10^3^—the conversion factor for changing units from J*mm^−2^ to kJ*m^−2^.

### 2.8. Three-Point Bending

Three-point flexural tests (PN-EN ISO 178) [28] were conducted with a ZwickRoell RetroLine universal tester (Ulm, Germany) driven by *testXpert II*. The specimen width *b* and minimum thickness *h_min_* were measured to 0.01 mm; the span followed the standard. After a 0.1 N preload, the cross-head ran at 10 mm min^−1^ to determine the flexural modulus E_f_, and then at 50 mm min^−1^ to reach the maximum flexural stress σ_fM_. The software reported E_f_, σ_fM_, and the corresponding outer-surface strain ε_fM_.

### 2.9. Mechanical Properties Under Static Tensile Loading

Tensile testing (PN-EN ISO 527-1) [29] was carried out with a ZwickRoell 1435 universal tester (Ulm, Germany). Injection-molded type 1A dumbbells (≈10 mm gauge width) were measured to 0.01 mm at three random points; the lowest width *b* and thickness *h* were used. At least three specimens per formulation were tested. From the stress–strain curve, the software provided Young’s modulus E_mod_, maximum tensile stress (TS), strain at maximum force E_Fmax_, and elongation at break E_b_.

### 2.10. VICAT Softening Temperature

The Vicat softening temperature (VST) was determined in accordance with PN-EN ISO 306 [30] with a DVicat/3/250/FA three-station tester (ZwickRoell, Ulm, Germany) filled with SILOIL silicone oil. Injection-molded bars of a 4 mm nominal thickness were placed horizontally on the supports and loaded with 50 N (method A50). The temperature was raised from 30 °C to 170 °C at 2 K min^−1^ (120 K h^−1^) until a 1 mm needle penetration was reached. Three parallel determinations were performed simultaneously for each material.

### 2.11. Melt Flow Index (MFI)

Melt-mass and melt-volume flow rates (MFR/MVR) were measured in accordance with PN-EN ISO 1133-1 with a Melt-Flow Plus tester (Karg Industrietechnik, Krailling, Germany). The tests were run at 190 °C under a 2.16 kg load after a 240 s pre-heat. Approximately 4 g of granulate was charged to the cylindrical barrel; the capillary die had a 2.095 mm diameter and 8 mm length, and piston travel for the timed cut-off was 25.4 mm. The instrument automatically recorded the extrusion time over the set stroke and reported the MFR (g 10 min^−1^) and the corresponding MVR (cm^3^ 10 min^−1^).

### 2.12. Thermal Analysis (DSC/TGA)

Thermogravimetric analysis (TGA) was carried out with a TGA/DSC 1 STAR System (Mettler-Toledo, Greifensee, Switzerland). Roughly 10 mg of each composite, placed in aluminum crucibles with perforated lids, was first heated from 25 °C to 600 °C at 10 °C min^−1^ under flowing argon (50 mL min^−1^) to monitor the thermal decomposition of the polypropylene matrix and the biofiller. Immediately afterward, the purge gas was switched to air (50 mL min^−1^), and the run continued to 900 °C, enabling complete oxidation of the char residue and, thus, quantification of the inorganic fraction.

Differential scanning calorimetry (DSC) was performed with the same instrument using a three-segment protocol at a constant rate of 10 °C min^−1^. Samples were first heated from −50 °C to 200 °C to erase their thermal history and record their initial melting behavior, then cooled back to −50 °C to capture crystallization, and finally reheated to 200 °C to determine the glass transition temperature, degree of crystallinity, and equilibrium melting enthalpy.

### 2.13. Rheological Properties

Rheological measurements were carried out on a rotational ARES-G2 (TA Instruments, New Castle, DE, USA) equipped with a torque-rebalance (TR) motor/sensor and stainless-steel parallel plates (Ø 25 mm). Test specimens, pressed from weighed portions of granulate in a heated hydraulic press, were punched into 25 mm discs of a ~2 mm thickness and conditioned for ≥24 h before measurement.

To evaluate the temperature dependence of melt viscosity, each disc was clamped between the plates, pre-heated for 30 s at 130 °C, and then subjected to a linear heating ramp of 5 °C min^−1^ to 280 °C under a constant shear rate. The instrument, operated in flow mode, continuously logged the instantaneous temperature and apparent viscosity η.

A fresh disc was then analyzed isothermally at 200 °C to obtain the viscosity–shear-rate profile. After a 60 s thermal equilibration, the rheometer increased the shear rate stepwise from the minimum to the maximum values available for the material, recording the corresponding apparent viscosity η at each γ.

### 2.14. Surface Wettability (WCA)

Static water contact angles were measured with a goniometer OCA 15 EC (DataPhysics Instruments, Filderstadt, Germany). A 3 µL drop of de-ionized water was deposited on the surface of each bar, after which a rectangular region of interest was drawn around the droplet. The software automatically traced the contour and calculated the left- and right-hand contact angles; their average was taken as the value for that spot. Ten such measurements, made at different locations, were recorded for every formulation.

## 3. Results and Discussion

### 3.1. Thermal Properties

#### 3.1.1. Vicat Softening Temperatures (VST)

**Figure 1:**
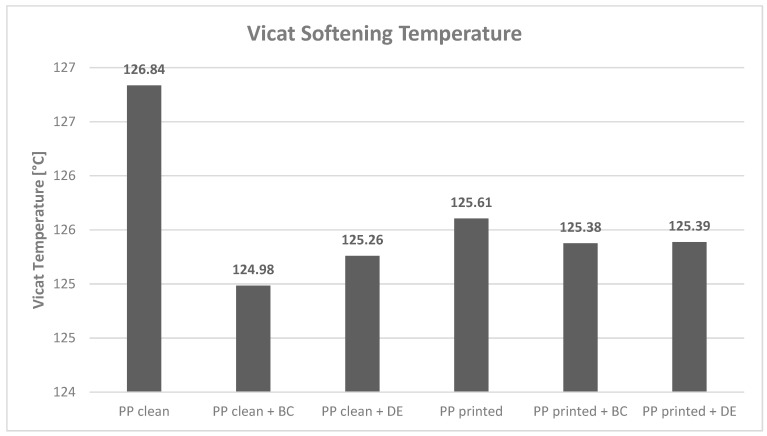
Vicat softening temperature plot for polypropylene specimens: neat PP, PP with 4.8 wt% biochar (BC) or diatomaceous earth, and corresponding variants with a printed ink layer.

Figure 1 presents the Vicat softening temperatures (VST) of the reference polypropylene (PP) samples—both unprinted and printed—as well as composites modified with 4.8 wt% of biochar or diatomaceous earth, including their respective printed variants. The VST values ranged between 124.98 °C and 126.84 °C. A slight reduction in the softening temperature was observed in the printed PP sample compared with its unprinted counterpart, from 126.84 °C to 125.61 °C. This effect may have resulted from the presence of dyes and low-molecular-weight ink components, such as solvents and resins, which can act as plasticizers by decreasing the order of the polymer chains and enhancing their segmental mobility at elevated temperatures [31]. This observation is consistent with the literature data indicating that foreign phases in polyolefins reduce crystallinity and, consequently, lower the VST [31].

During recycling, the thermal degradation of ink components becomes particularly relevant. Nitrocellulose (NC), commonly used as a binder in flexographic inks for PP packaging, decomposes and hydrolyzes in the 160–210 °C range, overlapping with PP processing temperatures [31]. The resulting low-molecular-weight degradation products may further plasticize the composite, reducing its softening temperature. Additionally, trace amounts of metals present in pigments may catalyze PP chain degradation via free radical formation [31], contributing to the observed VST decrease.

The incorporation of biochar caused a moderate decrease in the VST relative to the unmodified PP. In unprinted films, the reduction was approximately 1.9 °C, while in printed films, it was only 0.2 °C. This effect can be explained by the dominance of amorphous-phase segmental motion around the softening point, where the low filler content does not form a sufficient mechanical support network [32]. Biochar may also displace a portion of the crystalline PP phase, leading to a slight reduction in the average VST.

Diatomaceous earth exhibited a similar trend. When introduced at 4.8 wt%, it resulted in a minor VST decrease of approximately 1.6 °C in unprinted films and 0.2 °C in printed ones. According to the literature, significant increases in the VST for DE-filled composites occur only at higher filler contents, typically above 15 wt% [33]. This phenomenon may be attributed to matrix dilution and potential interactions between filler surface functionalities and polymer chains [34].

#### 3.1.2. Differential Scanning Calorimetry (DSC) Analysis

**Figure 2:**
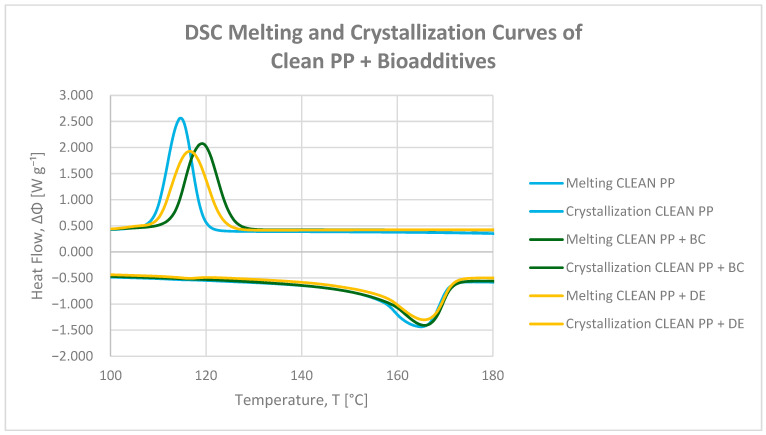
DSC melting (endo peak) and crystallization (exo peak) curves of neat polypropylene and PP composites with 4.8 wt% biochar (BC) and diatomaceous earth (DE).

**Figure 3:**
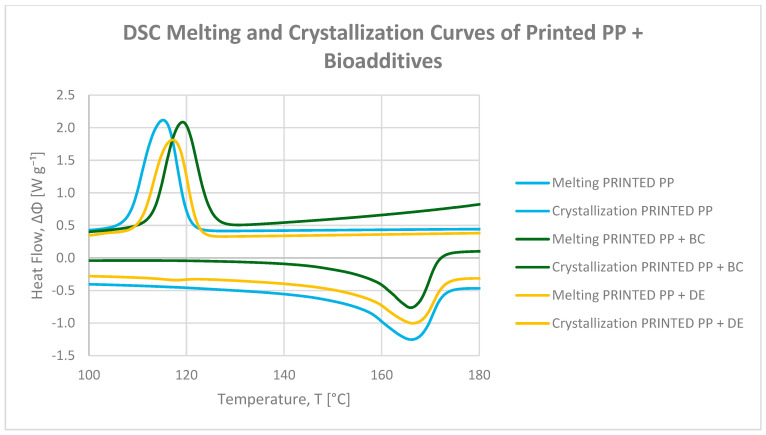
DSC melting (endo peak) and crystallization (exo peak) curves of printed polypropylene and PP composites with 4.8 wt% biochar (BC) and diatomaceous earth (DE).

DSC melting and crystallization curves for neat and printed polypropylene (PP), as well as composites containing 5 wt% biochar (BC) or diatomaceous earth (DE), are presented in Figure 2 and Figure 3. The values of crystallization temperatures (Tc onset, Tc peak, and Tc endset) and crystallization enthalpy of composites are summarized in Table 2 and Table 3. The thermal profiles allow assessment of phase transition temperatures and crystallinity changes resulting from biofiller incorporation, while considering their limited impact on practical heat resistance (VST).

Both BC and DE exhibited nucleating effects, as evidenced by higher crystallization onset temperatures. Biochar proved particularly effective, raising Tc onset by 6.01 °C in neat PP and 4.67 °C in printed PP [32]. Diatomaceous earth induced smaller increases of 4.11 °C and 1.86 °C, respectively, suggesting moderate nucleation efficiency. This difference may stem from the better surface compatibility of carbonaceous particles with polyolefin chains, while the siliceous nature of DE limits nucleation due to weaker interfacial interactions [35].

Although the crystallization onset temperature increased, a simultaneous decrease in crystallization enthalpy (∆Hc) was observed upon filler addition. This indicates that while nucleation is enhanced, the overall degree of crystallinity may actually decrease due to spherulite growth inhibition and restricted chain mobility near filler surfaces [36]. Therefore, the apparent increase in Tc should not be interpreted as enhanced structural order, but rather as a filler-induced shift in nucleation and crystallization kinetics. In printed PP, the enthalpy reduction was more pronounced (8.5–12.7%), highlighting the additional impact of residual printing ink on limiting chain mobility and crystal growth [31].

Minimal changes in melting temperatures indicate that the structural integrity of the crystalline PP phase was largely preserved. However, it should be noted that this thermal stability refers specifically to the crystalline phase and does not necessarily translate into improved heat resistance under load, as reflected by the slight decrease in the Vicat softening temperature (VST) observed for both BC and DE composites.

Overall, biochar is a more effective nucleating additive than diatomaceous earth, promoting higher crystallization temperatures, but the concurrent decrease in ∆Hc indicates that the total crystallinity is reduced. Diatomaceous earth, although less effective in raising Tc onset, supports more uniform spherulite growth. Residual printing ink further modulates crystallization behavior, particularly in reducing enthalpy, confirming a parameter-dependent thermal response consistent with TGA and VST observations.

### 3.2. Thermogravimetric Analysis

**Figure 4:**
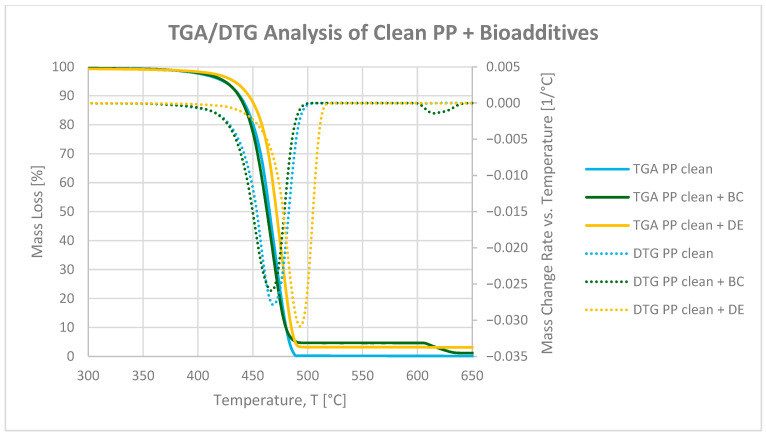
TGA and DTG curves of neat polypropylene and PP composites with 4.8 wt% biochar (BC) and diatomaceous earth (DE).

**Figure 5:**
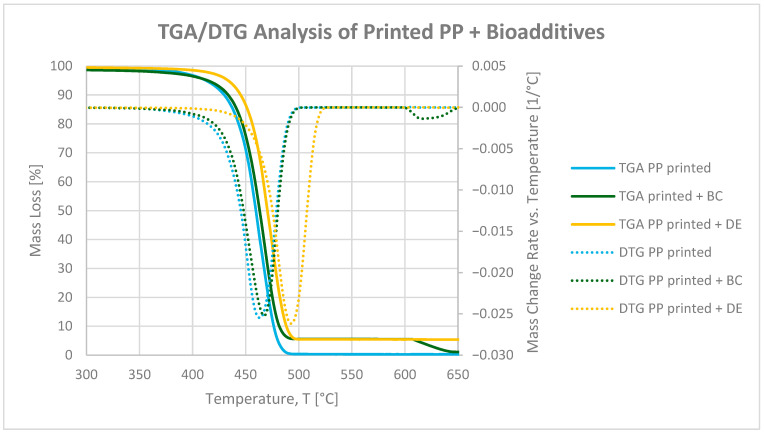
TGA and DTG curves of printed polypropylene and PP composites with 4.8 wt% biochar (BC) and diatomaceous earth (DE).

Figure 4 and Figure 5 present thermogravimetric (TGA) and derivative thermogravimetric (DTG) curves for neat and printed polypropylene (PP), as well as composites with 4.8 wt% biochar (BC) or diatomaceous earth (DE). The analysis was performed in inert gas (Ar) from 25 to 900 °C, followed by oxidation in air until complete degradation.

The addition of 4.8 wt% BC had a negligible impact on the initial thermal degradation of PP, with similar 5% weight loss temperatures (T_5_) observed for neat PP (425 °C) and the BC composite (424 °C), as summarized in Table 4. In contrast, DE slightly delayed the onset of degradation, increasing T_5_ to 433 °C. This effect can be attributed to the siliceous structure of DE, which reflects heat and limits gas diffusion, acting as a thermal barrier [37].

Although BC does not shift T_5_, its porous structure may contribute to stabilization at later degradation stages, as indicated by a shift in the temperature of the maximum degradation rate (TDTGMax) and increased char residue [32]. In the oxidative phase (600–900 °C), the residual mass at 900 °C (R_900_) increased from 0.29% (neat PP) to 1.19% (with BC) and 3.17% (with DE), due to incomplete combustion of aromatic BC fractions and the presence of inert silica. The DTG curves indicate minor retardation of the main pyrolytic degradation step, with the maximum degradation temperature shifting from 469 °C (neat PP) to 467 °C with BC and 475 °C with DE [38,39].

In printed PP, the residual ink lowered T_5_ from 425 °C (neat) to 413 °C, likely due to pigments, resins, and volatiles initiating early degradation. Addition of BC slightly increased T_5_ to 415 °C, whereas DE again raised it to 433 °C, indicating that the filler’s barrier effect is retained even with the ink layer. The DTG degradation peak shifted from 463 °C (printed PP) to 467 °C (with BC) and 473 °C (with DE), confirming a modest delay of the main pyrolytic degradation step. These effects are attributed to free radical adsorption on BC and formation of a protective carbon–silica layer by DE [39,40].

Note: While DE slightly raises the T_5_ value, indicating a minor delay in thermal degradation, practical thermal resistance measured by the Vicat softening temperature (VST) decreased with both fillers. Therefore, the term “thermal stability enhancement” should be interpreted specifically in terms of thermal degradation behavior, not mechanical or processing-related heat resistance.

### 3.3. Mechanical Properties

#### 3.3.1. Hardness

**Figure 6:**
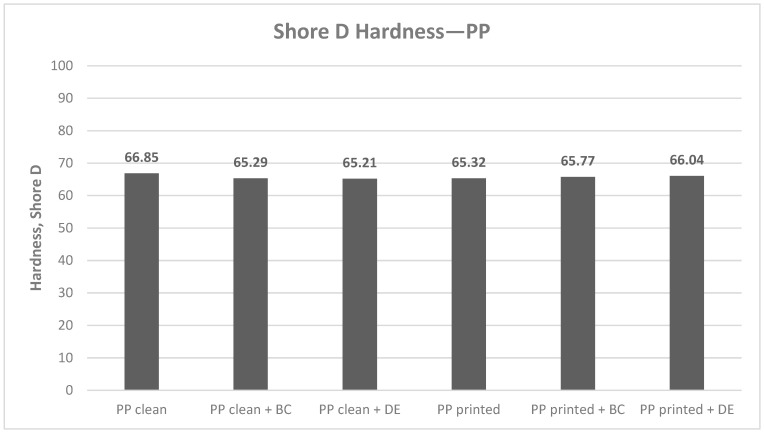
Average Shore D hardness values for polypropylene samples: reference materials (neat PP and printed PP) and composites with 4.8 wt% biochar (BC) and diatomaceous earth (DE).

The chart below (Figure 6) presents the average Shore D hardness values obtained for all polypropylene variants, including reference materials (neat PP and printed PP) and composites containing 4.8 wt% biochar (BC) or diatomaceous earth (DE).

The Shore D hardness measurements of polypropylene (PP) composites containing 4.8 wt% biochar (BC) or diatomaceous earth (DE) revealed only slight differences attributed to the type of filler and the presence of the flexographic ink layer. Across all tested variants, the changes remained within ±1.64 ShD, corresponding to a maximum deviation of ±2.5%, indicating that the influence of bioadditives and ink residues on surface hardness is minimal.

In unprinted PP, BC reduced hardness by 1.56 ShD (−2.33%), and DE by 1.64 ShD (−2.45%). These values fall within the typical range of variation reported for polymeric systems and are considered negligible in practical applications, where mechanical property deviations below 10% are generally acceptable.

Flexographic printing led to a slight decrease in PP hardness by 1.53 ShD (−2.29%). Interestingly, when biofillers were introduced to the printed samples, this effect was partially mitigated: BC increased hardness by 0.45 ShD (+0.69%) and DE by 0.72 ShD (+1.10%). Although this suggests a minor reinforcing effect in the presence of ink, the magnitude of these changes is too low to significantly affect the overall mechanical performance of the material.

#### 3.3.2. Impact Strength

**Figure 7:**
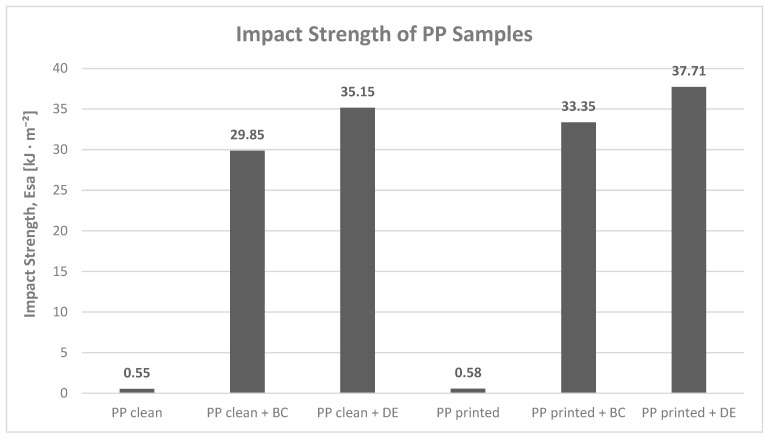
Charpy impact energy (E_sa_) of polypropylene samples: reference materials and those modified with 4.8 wt% biochar (BC) or 4.8 wt% diatomaceous earth (DE).

Impact strength testing of polypropylene composites containing 4.8 wt% biochar (BC) or diatomaceous earth (DE) revealed a clear change in fracture behavior (Figure 7). The addition of biofillers did not lead to a general “improvement” in toughness, but rather to a fundamental shift in fracture mode, from ductile deformation with very low energy absorption to complete brittle failure associated with much higher absorbed energy. The absorbed energy increased 54- to 65-fold compared with unmodified PP, which should be interpreted as a change in the fracture mechanism rather than a straightforward enhancement of mechanical performance. This highlights the significant influence of the fillers on crack propagation and damage mechanisms within the material.

Neat and printed PP samples exhibited typical ductile fracture, with absorbed energy around 0.55–0.58 kJ/m^2^, and no complete failure [2]. In contrast, composites with BC absorbed 29.9 kJ/m^2^ (neat PP) and 33.3 kJ/m^2^ (printed PP), while DE composites reached 35.1 kJ/m^2^ and 37.7 kJ/m^2^, respectively. In all cases, full specimen fracture occurred.

The increased brittleness is primarily attributed to stress concentration around rigid filler particles, which promotes microcrack initiation [41]. This effect is intensified by the irregular shape and surface morphology of biofillers. DE showed greater energy absorption than BC—by 17.7% in neat PP and 13.1% in printed PP—likely due to its porous silica structure, which enhances interfacial interactions and energy dissipation [42].

### 3.4. Three-Point Bending

**Figure 8:**
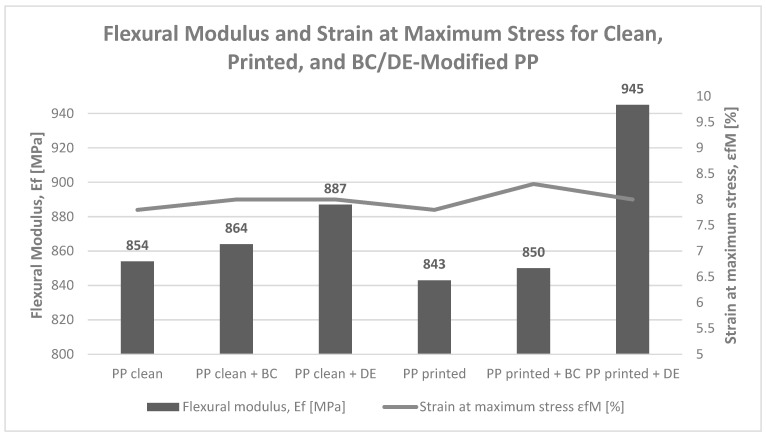
Flexural modulus (Ef) and strain at maximum stress (εfM) for polypropylene (PP), both unprinted and printed, and their composites containing 4.8 wt% biochar (BC) or diatomaceous earth (DE).

Three-point bending tests of polypropylene (PP) composites containing biochar (BC) and diatomaceous earth (DE) indicate a selective influence of fillers on material stiffness (Figure 8). Diatomaceous earth exhibited a clear stiffening effect, increasing the flexural modulus from 854 MPa (neat PP) to 887 MPa (with DE) and up to 945 MPa in the presence of a flexographic ink layer. In contrast, biochar had a negligible effect, suggesting limited interaction with the PP matrix under static loading conditions. Unlike in Charpy impact testing, all specimens remained structurally intact under bending, confirming ductile behavior at lower deformation rates.

DE demonstrated superior stiffening capability, consistent with the literature on siliceous mineral fillers. For neat PP, DE increased the flexural modulus by 3.9% (854 → 887 MPa), and by 12.1% in printed PP (843 → 945 MPa), confirming its effectiveness [33]. In comparison, BC-modified composites exhibited only minor changes—864 MPa for unprinted and 850 MPa for printed samples—remaining close to the reference material.

The low stiffening efficiency of BC can be attributed to its lower modulus relative to mineral fillers and poor stress transfer due to limited filler–matrix interactions. The literature reports significant improvements in stiffness only at higher BC contents (20–30 wt%), with up to 100% increases in tensile and 140% in flexural modulus [43].

Notably, a slight synergistic effect was observed in the printed DE composite, which achieved the highest modulus (945 MPa). This may result from enhanced interfacial adhesion between the ink layer and DE particles, promoting more efficient stress transfer. No such effect was observed in BC-based systems.

### 3.5. Tensile Strength and Elongation at Break

**Figure 9:**
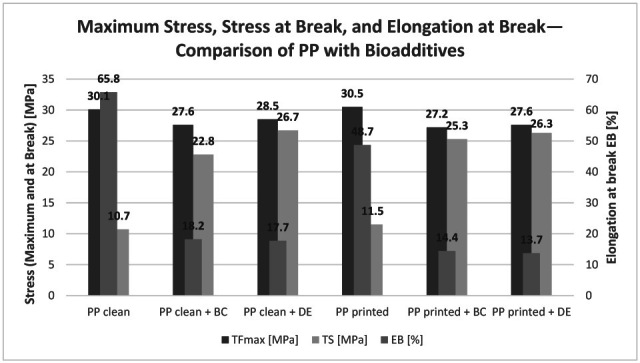
Maximum tensile force (TFmax), tensile strength at break (TS), and elongation at break (EB) of the tested PP samples.

**Figure 10:**
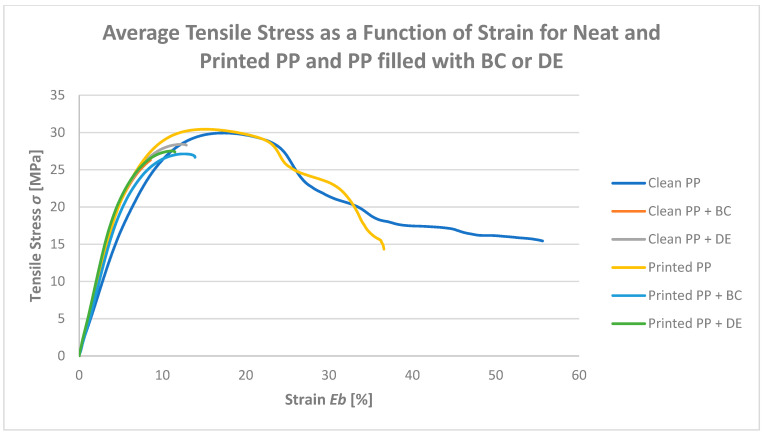
Stress–strain curves of the investigated materials.

Static tensile testing was conducted to evaluate the effects of bioadditives and flexographic printing on the mechanical properties of polypropylene composites (Figure 9). Three key parameters were analyzed: maximum tensile force (TFmax), tensile strength at break (TS), and elongation at break (EB). To provide a clearer representation of the results, stress–strain curves (Figure 10) of the tested materials were also prepared.

For both reference regranulates (without biofillers), TFmax values were nearly identical—30.1 MPa for neat PP and 30.5 MPa for printed PP—indicating that the ink does not affect yield strength. However, elongation at break decreased from 65.8% to 48.7%, suggesting that pigments and resins in the ink act as micro-stress concentrators, limiting plastic deformation. Simultaneously, TS slightly increased from 10.7 MPa to 11.5 MPa, showing that printed samples fracture at higher stresses but lower strains.

The incorporation of only 4.8 wt% biochar (BC) or diatomaceous earth (DE) markedly modified the deformation behavior. TFmax dropped by 8–11% to around 27–28 MPa, regardless of printing. At the same time, TS more than doubled to 22–27 MPa, while EB sharply declined to 14–18%, reflecting a transition from ductile to brittle fracture. This apparent contradiction—lower maximum tensile force but higher tensile strength at break—indicates that the fillers change the fracture mechanism by reducing the ability of the polymer to sustain plastic deformation, while simultaneously allowing the material to withstand higher localized stresses before final failure. Therefore, the observed “improvement” concerns specific strength-related parameters, but it cannot be interpreted as a universal enhancement of mechanical performance. Thus, the fillers substantially enhanced strength-related parameters (higher TS), but at the cost of ductility (lower EB), which is a typical trade-off observed in particle-reinforced composites.

These findings align with the literature reports: rigid filler particles restrict polymer chain mobility and create stress concentration zones that facilitate crack initiation with minimal necking. Similar reductions in EB and increases in TS were observed in PP and PLA composites containing biochar and diatomaceous earth [17,32].

Importantly, the presence of the flexographic print in regranulates did not alter the overall effect of the biofillers. Printed and non-printed composites with BC or DE exhibited comparable TFmax, TS, and EB values.

### 3.6. Rheological Properties

#### 3.6.1. Melt Flow Index

**Figure 11:**
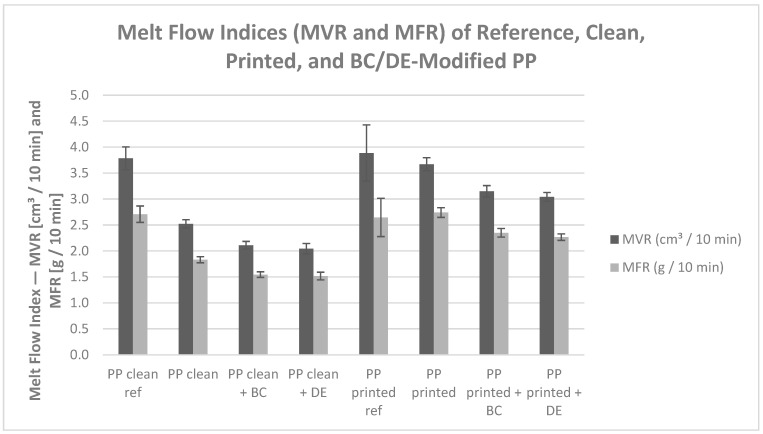
Comparison of MVR (cm^3^/10 min) and MFR (g/10 min) flow rates for reference polypropylene, after regranulation, and composites containing 4.8 wt% biochar (BC) or diatomaceous earth (DE).

The melt flow behavior of PP composites with biochar and diatomaceous earth revealed interactions among three key factors: BOPP film regranulation, dispersed flexographic ink residues, and biobased fillers (Figure 11). These elements jointly influence the rheological behavior of the melt, affecting processability in a filler-specific manner.

Regranulation of clean BOPP film leads to a significant increase in melt viscosity, as evidenced by a decrease in the MVR from 3.79 to 2.52 cm^3^/10 min and the MFR from 2.71 to 1.83 g/10 min (reductions of 33% and 32%, respectively) in Table 5. This effect is attributed to thermo-mechanical degradation of polypropylene, including molecular changes, such as partial crosslinking or recrystallization, which hinder polymer flow.

Flexographic ink improves melt flow, with the MVR and MFR increasing to 3.67 cm^3^/10 min and 2.74 g/10 min, respectively, about 45% higher than in unprinted re-granulate. This is due to the presence of low-molecular-weight resins, pigments, and solvent residues in the ink, which act as internal lubricants by increasing chain mobility [44]. Quantitatively, flexographic ink restores flow parameters nearly to the level of neat PP, reducing the viscosity losses from regranulation by ~90%, which demonstrates its strong lubricating action.

Biochar and diatomaceous earth have opposing effects on viscosity, highlighting a filler-dependent processability.

Biochar significantly lowers viscosity, particularly in ink-containing systems. At 200 °C, the viscosity of PP + ink + BC drops to 380.9 Pa·s, a 78.5% decrease versus PP + ink (1772.2 Pa·s). This pronounced effect may stem from the lubricating nature of biochar particles and their disruption of PP crystallinity.

Diatomaceous earth increases viscosity in unmodified systems (PP + DE: 3741.5 Pa·s, +97.1% relative to pure PP) but reduces it when ink is present (PP + ink + DE: 1106.1 Pa·s, −37.6% compared with PP + ink). This behavior reflects high porosity and surface interactions modulated by ink components.

Thus, “processability” must be considered separately for each filler: BC improves flow via viscosity reduction, allowing lower processing temperatures, whereas DE can require higher pressures or temperatures in clean systems, but ink mitigates this effect in printed composites.

#### 3.6.2. Determination of Viscosity as a Function of Temperature (130–280 °C)

**Figure 12:**
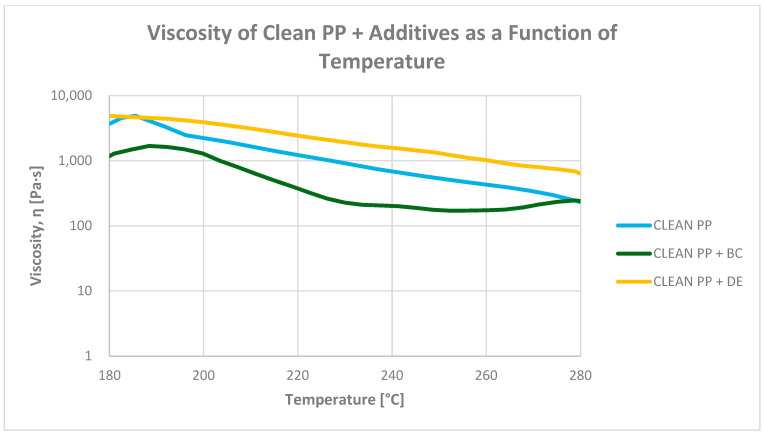
Dependence of re-granulate viscosity on temperature for polypropylene without additives and PP composites with 4.8 wt% biochar (BC) and diatomaceous earth (DE).

**Figure 13:**
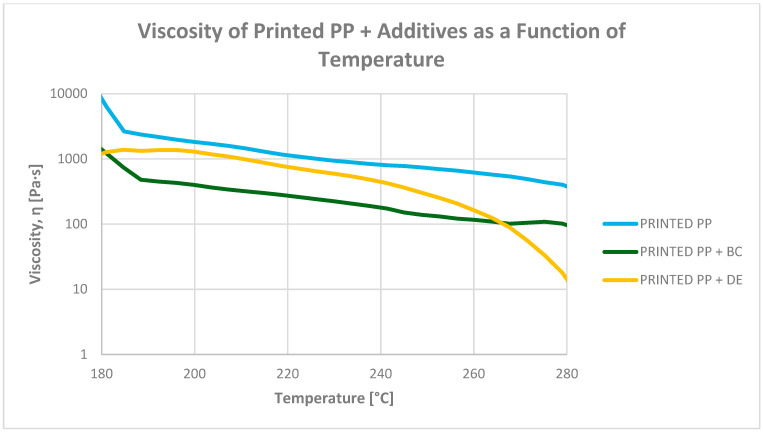
Dependence of re-granulate viscosity on temperature for printed polypropylene and biochar (BC) and diatomaceous earth (DE) composites.

Rheological analysis of polypropylene composites revealed complex viscosity changes arising from the interplay of thermal degradation, plasticization, and filler–matrix interactions (Figure 12 and Figure 13). Flexographic ink, dispersed throughout the granulate, acts as an effective internal plasticizer, reducing viscosity by 62–70% at 200 °C compared with systems without ink. For example, the viscosity of re-granulated PP drops from 1772.2 Pa·s to 664.5 Pa·s when ink is present (−62.5%), quantitatively confirming the ink’s strong effect on melt flow.

Biochar significantly reduces viscosity across the entire tested temperature range, particularly in ink-containing systems. At 200 °C, the viscosity of the PP + ink + BC composite drops to 380.9 Pa·s, representing a 78.5% decrease versus PP + ink (1772.2 Pa·s). This pronounced effect may stem from the lubricating nature of biochar particles and their disruption of PP crystallinity.

In contrast, diatomaceous earth (DE) increases viscosity in unmodified systems but reduces it when ink is present. For PP + DE (without ink), viscosity rises to 3741.5 Pa·s, a 97.1% increase relative to pure PP. However, in the PP + ink + DE system, viscosity drops to 1106.1 Pa·s, reflecting a 37.6% reduction compared with PP + ink. This behavior is likely due to the high porosity and surface area of DE, which promote internal friction in clean systems. In ink-containing composites, interactions between DE surface groups and ink components may lead to hybrid structures with altered flow characteristics.

These rheological differences directly influence processing requirements. Composites with BC, due to their lower viscosity, can be processed at reduced temperatures, enhancing energy efficiency. Conversely, DE-based systems—especially without ink—exhibit high viscosity, requiring increased injection pressures or higher temperatures to maintain adequate melt flow and mold filling.

#### 3.6.3. Determination of Viscosity as a Function of Shear Rate (at 200 °C)

**Figure 14:**
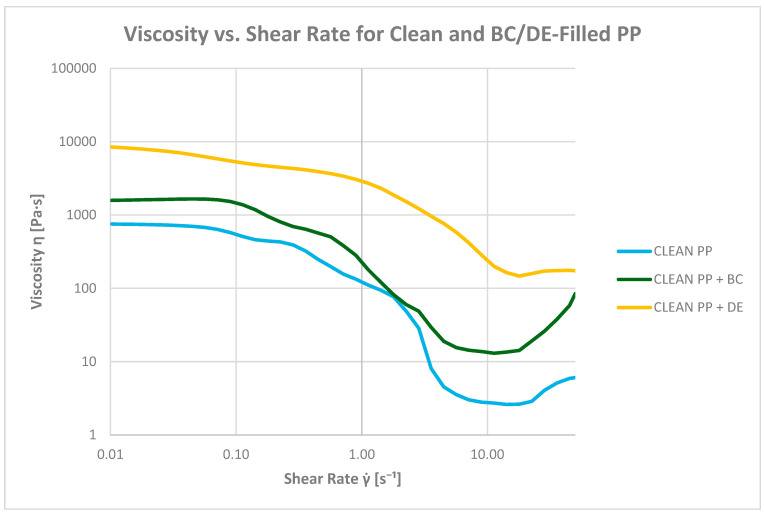
Dependence of composite viscosity on shear rate for pure polypropylene (PP) and bioadditives.

**Figure 15:**
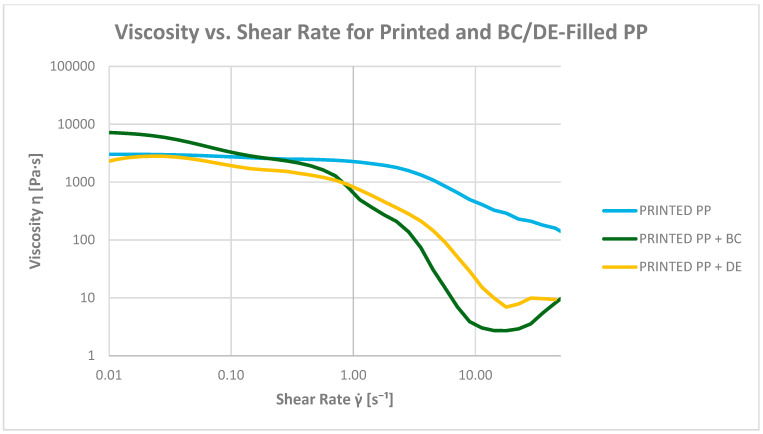
Dependence of composite viscosity on shear rate for printed polypropylene (PP) and bioadditives.

All PP composites exhibit shear-thinning behavior at 200 °C, facilitating melt flow under dynamic conditions (Figure 14 and Figure 15). The viscosity trends confirm the distinct rheological contributions of BC and DE, which should not be generalized under a single definition of processability.

At a low shear rate (0.1 s^−1^), all materials show Newtonian plateau behavior. Neat PP displays a baseline viscosity of 473.37 Pa·s. Adding biochar increases this to 1300.06 Pa·s (2.75×), attributed to physical interactions and network formation limiting polymer chain mobility. Diatomaceous earth has a more pronounced effect, increasing viscosity to 4830.83 Pa·s (10×), due to its high surface area and porosity promoting strong matrix interactions.

Printed PP shows a higher baseline viscosity (2609.20 Pa·s), likely due to ink components or molecular structure modifications. Adding biochar raises viscosity slightly (3111.70 Pa·s), while DE surprisingly reduces it to 1763.54 Pa·s, possibly indicating reduced compatibility or altered dispersion in the ink-modified matrix.

At 1 s^−1^, viscosity decreases across all samples, confirming pseudoplastic behavior. Neat PP drops to 107.73 Pa·s (−77%), and PP + BC to 148.85 Pa·s. The PP + DE composite remains the most viscous (2270.12 Pa·s), although shear sensitivity becomes more evident at this rate.

For printed PP, the viscosity is 2055.31 Pa·s at 1 s^−1^ (−21%). With BC, it decreases significantly to 495.93 Pa·s, and with DE, to 653.01 Pa·s. In shear rate-dependent tests, the presence of ink modifies the apparent viscosity profiles, reducing the viscosity of DE-containing composites by 37.6% and of BC-containing composites by 78.5% relative to PP + ink alone, highlighting filler–ink interactions.

At 10 s^−1^, all composites show a steep decline in viscosity. Neat PP reaches 2.65 Pa·s (−99.4%). Adding BC increases viscosity to 13.34 Pa·s (5×), while DE raises it to 135.67 Pa·s (51×), highlighting its strong influence even under high shear.

Notably, the PP + ink + BC composite displays nearly Newtonian-like flow at 3.03 Pa·s—similar to neat PP—suggesting synergistic interactions between biochar and ink, leading to plasticizing-like effects. For comparison, printed PP reaches 289.94 Pa·s, while PP + ink + DE drops to 10.57 Pa·s.

### 3.7. WCA—Water Contact Angle Measurements

**Figure 16:**
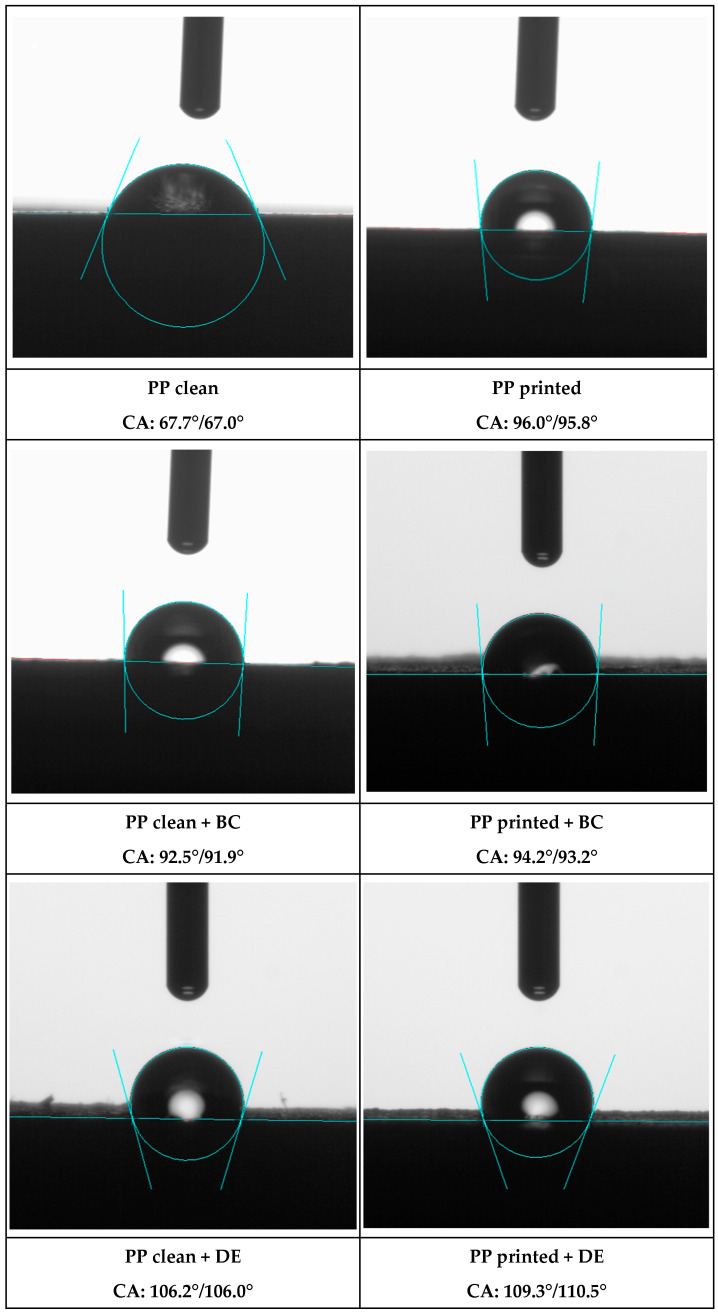
Average water contact angle (Θ) for printed and unprinted PP composites containing 4.8 wt% biochar (BC) or diatomaceous earth (DE).

Water contact angle measurements reveal significant changes in surface characteristics of PP composites with biochar and DE (Figure 16). Base regranulates show a transition from slightly hydrophilic to distinctly hydrophobic behavior, particularly in the presence of flexographic ink. The ink components, dispersed throughout the matrix, strongly reduce surface wettability. Additionally, each biofiller influences final surface properties through its inherent chemistry and particle morphology.

Neat PP re-granulate exhibits a hydrophilic surface (WCA = 67.33°), significantly lower than the typical literature values for unmodified PP (94–110°) [45]. This deviation likely results from surface oxidation, increased roughness, or residual contaminants introduced during BOPP film reprocessing.

Incorporation of biofillers induces a clear shift toward hydrophobicity. Biochar increases WCA to 92.43° (+37.3%), while diatomaceous earth further elevates it to 106.73° (+58.5%). These changes are consistent with prior findings on mineral and carbon-based fillers, where increased hydrophobicity is attributed to surface topography modification and functional group presence.

Flexographic ink alone significantly enhances PP hydrophobicity, raising the WCA from 67.33° to 95.37° (+41.7%), due to low-polarity compounds such as organic pigments, resins, and hydrocarbon-based additives [31]. This increase corresponds to a shift of 28.04°, quantitatively demonstrating the strong effect of ink residues on wettability, which dominates over biofiller contributions in printed composites.

In printed composites, the impact of biofillers is reduced. Biochar shows a negligible additional effect (WCA = 95.31°), while DE increases it to 109.51° (+14.8%). The diminished influence of fillers is likely due to the dominant surface-modifying effect of ink components, which overshadows the fillers’ contribution to wettability.

## 4. Conclusions

The reprocessing of BOPP films slightly reduced the Vicat softening temperature (VST) due to thermo-mechanical polymer degradation. Biochar (BC) and diatomaceous earth (DE), added at 4.8 wt%, did not markedly improve thermal resistance under load, but both acted as nucleating agents, increasing crystallization onset temperatures and reducing enthalpy, indicating the formation of finer crystalline structures. BC showed a stronger nucleating effect, while DE promoted more uniform spherulite growth. However, in both cases, the overall crystallinity decreased, which may negatively affect long-term dimensional stability.

Thermogravimetric analysis revealed filler-dependent modifications of thermal stability. DE slightly increased the onset of degradation (T_5_), whereas BC delayed the maximum degradation rate (TDTGMax). Both fillers, however, reduced the Vicat softening temperature, highlighting that thermal effects should be considered parameter-specific rather than a general improvement. Residual mass increased, particularly with DE. Despite these parameter-specific improvements, the reduction of VST emphasizes that the fillers do not enhance practical heat resistance during processing or application.

Mechanical tests confirmed a clear trade-off between strength and ductility. Compared with virgin BOPP, the tensile properties of regranulates decreased significantly, especially elongation at break. While tensile strength at break (TS) increased in filled systems, maximum tensile force (TFmax) and elongation at break (EB) decreased, reflecting a transition from ductile to brittle fracture. This increased brittleness, although linked with higher stress-bearing capacity, limits the toughness and reliability of the composites in demanding applications. DE more effectively improved stiffness and impact strength, partly compensating for brittleness introduced by the ink. In contrast, BC led to reduced elongation and lower strength retention. Overall, the mechanical performance gains are therefore accompanied by non-negligible drawbacks in ductility and fracture resistance.

Rheological studies showed shear-thinning behavior in all systems. BC markedly reduced viscosity, especially in ink-containing composites, suggesting synergistic plasticizing effects between biochar and ink components. DE increased viscosity in neat systems but less so in printed composites, and its rheological response was more sensitive to shear rate. Thus, BC improves processability, while DE may hinder it in ink-free systems due to higher viscosity requirements, reducing its technological attractiveness at low filler loadings.

Surface wettability measurements demonstrated a transition from hydrophilic to hydrophobic behavior. Flexographic ink strongly enhanced hydrophobicity, and DE further increased water contact angle, while BC had a limited effect in printed composites due to the dominant role of low-polarity ink components.

## Figures and Tables

**Table 1:** Formulation of PP composites containing biochar (BC) or diatomaceous earth (DE), expressed per 100 parts by weight of PP.

Sample	PP [pbw]	Biochar (BC) [pbw]	Diatomaceous Earth (DE) [pbw]	Biofiller [wt %]
PP clean	100	-	-	0
PP clean + BC	100	5	-	4.8
PP clean + DE	100	-	5	4.8
PP printed	100	-	-	0
PP printed + BC	100	5	-	4.8
PP printed + DE	100	-	5	4.8

**Table 2:** Crystallization temperatures (Tc onset, Tc peak, and Tc endset) and crystallization enthalpy of neat/printed PP and PP composites with biochar (BC) or diatomaceous earth (DE), determined from the DSC cooling segment.

Sample	Tc_onset_ [°C]	Tc_peak_ [°C]	Tc_endset_ [°C]	∆Hc [J/g]
PP clean	119.72	116.14	109.61	83.42
PP clean + BC	125.73	120.07	113.2	81.35
PP clean + DE	123.83	117.61	110.08	81.22
PP printed	120.65	116.05	108.24	84.46
PP printed + BC	125.32	120.04	113.16	77.32
PP printed + DE	122.51	117.84	110.48	73.77

**Table 3:** Melting temperatures (Tm onset, Tm peak, and Tm endset) and melting enthalpy of neat/printed PP and PP composites with biochar (BC) or diatomaceous earth (DE), determined from the DSC second-heating segment (main melting transition).

Sample	Tm_onset_ [°C]	Tm_peak_ [°C]	Tm_endset_ [°C]	∆Hm [J/g]
PP clean	153.24	163.88	170.47	−72.09
PP clean + BC	153.26	164.96	171.15	−73.42
PP clean + DE	152.94	164.93	171.34	−64.19
PP printed	152.06	165.56	172.46	−69.26
PP printed + BC	154.84	165.63	171.81	−62.72
PP printed + DE	153.05	166.07	172.81	−62.21

**Table 4:** Thermogravimetric parameters for neat PP and PP composites: temperature at 5% mass loss (T_5_), temperature at the DTG maximum (T_DTGMax_), mass loss between 25 and 600 °C (ΔM_25-600_), and residual mass at 900 °C (R_900_).

Sample	T_5_ [°C]	T_DTGMax_ [°C]	ΔM_25-600_ [%]	R_900_ [%]
PP clean	425	469	99.40	0.29
PP clean + BC	424	467	95.33	1.19
PP clean + DE	433	475	96.75	3.17
PP printed	413	463	99.68	0.35
PP printed + BC	415	467	94.40	1.15
PP printed + DE	433	473	94.51	5.46

**Table 5:** Melt-flow rates (MVR and MFR) of neat and printed PP and PP composites with biochar (BC) or diatomaceous earth (DE) at 190 °C/2.16 kg.

Sample	MVR (cm^3^/10 min)	SD MVR	MFR (g/10 min)	SD MFR
PP clean ref	3.79	±0.22	2.71	±0.16
PP clean	2.52	±0.08	1.83	±0.06
PP clean + BC	2.11	±0.07	1.55	±0.05
PP clean + DE	2.05	±0.10	1.52	±0.07
PP printed ref	3.89	±0.54	2.65	±0.37
PP printed	3.67	±0.13	2.74	±0.10
PP printed + BC	3.15	±0.11	2.35	±0.09
PP printed + DE	3.04	±0.09	2.27	±0.06

## Data Availability

The raw data supporting the conclusions of this article will be made available by the authors upon request.
